# The influence of heat exposure on birth and neonatal outcomes in Mombasa, Kenya: A pooled time series analysis

**DOI:** 10.1016/j.joclim.2024.100409

**Published:** 2025-01-15

**Authors:** Chloe Brimicombe, Debra Jackson, Aquinius Mungatia, Zeenat Sulaiman, Tobias Monthaler, Katharina Wieser, Ilona M Otto, HIGH Horizons study group, Stanley Luchters, Matthew Chersich, Gloria Maimela, Celeste Madondo, Shobna Sawry, Mags Beksinska, Lebohang Radebe, Ijeoma Solarin, Pascalia Munyewende, Chuansi Gao, Jakob Eggeling, Gunter Alce, Clara Heil, Nathalie Roos, Olof Stephansson, Claudia Hanson, Jeroen de Bont, Veronika Tirado, Anayda Portela, Jorn Toftum, Sohail Baloch, Jetina Tsvaki, Thabani Moronzie, Fortunate Machingura, Concilia Mutasa, Brian Mgondisi Sibanda, Tariro Chinozvina, Elizabeth Dangaiso, Jasper Maguma, Bongani Mutimutema, Veronique Filippi, Giulia Greco, Nasser Fardousi, Isabelle Lange, Giorgia Gon, Jo Borghi, Paul Lokubal, Cherie Part, Christo Hadjichristodoulou, Barbara Mouchtouri, Elina Kostara, Maria Kyritsi, Michalis Koureas, Fani Kalala, Chara Bogogiannidou, Ioanna Voulgaridi, Boris Kingma, Koen van der Sanden, Federica Nobile

**Affiliations:** a Wegener Centre for Climate and Global Change, University of Graz, Brandhofgasse 5, 8010 Graz, Austria; b MARCH Centre, London School of Hygiene and Tropical Medicine, Keppel St, London WC1E 7HT, United Kingdom; c School of Public Health, University of the Western Cape, Cape Town, South Africa; d Aga Khan Health Services, East Africa

**Keywords:** Extreme heat, Kenya, Perinatal, Maternal, Pregnancy, Wet bulb globe temperature, Universal thermal climate index, UTCI, Temperature, Climate change

## Abstract

**Introduction:**

The African continent has been identified as an area of high risk to increasing exposure of heat and has higher levels of social vulnerability. Heat exposure can lead to a rise in certain perinatal and maternal adverse health conditions. We explored the association of heat on seven perinatal and maternal health outcomes.

**Material and Methods:**

In this study, data is from Aga Khan University Hospital in Mombasa, Kenya. We evaluated the influence of heat exposure metrics on the outcomes of caesarean sections, low birth weight, low apgar score, preterm birth, stillbirth, assisted vaginal deliveries and long duration of stay in hospital. We carried out pooled time series regression using distributed-lag nonlinear models (lag 0–9 months).

**Results:**

We observed an increased odds of caesarean sections with heat exposure at lag 0 indicated by maximum daily Universal Thermal Climate Index (UTCI) between the 50th and the 95th percentile (relative risk 1.21 (1.01,1.46, 95 %CI)) and maximum daily temperature (1.25 (1.03,1.53)). There were increased odds of Low-Birth-Weight Births for lag 0 mean and maximum UTCI. We did not find any significant responses for Wet Bulb Globe Temperature (WBGT).

**Discussion and Conclusion:**

Our results show different risk responses for different heat exposure metrics for all perinatal and maternal health outcomes, significantly increasing for low-birth-weight births and caesarean sections. Further research is warranted for Kenya regarding maternal mortality and higher blood loss sometimes associated with caesarean deliveries. In addition, more research is needed on socioeconomics and heat exposure, especially in low– and middle income countries.

## 1 Introduction

The African continent has been identified as an area particularly at risk of adverse heat outcomes because of its susceptibility to the occurrence of heatwaves, as well as a highly vulnerable population [1]. One such country is Kenya, which is situated in the Horn of Africa region, in an area influenced by the Inter-tropical Convergence Zone (ITCZ) and East African Monsoon [2]. It has been previously found that there has been a statistically significant increase in heatwave days between 1986 and 2021 across the country as indicated using the HWMId (Heatwave Magnitude Index) [2].

Exposure to extreme heat can lead to adverse birth and maternal health outcomes [3]. In the context of Kenya, one study discussed the influence of heat on maternal health and wellbeing in rural Kilifi in coastal Kenya [4]. Pregnant women faced physical discomfort, skin conditions, and dehydration due to excessive perspiration impacting their overall wellbeing; and they had concerns about hypertension and adverse pregnancy outcomes[4]. Gendered division of labour exacerbated these challenges, as women continued strenuous household chores even in the heat, facing exhaustion and risking complications [4]. While sympathy was expressed, concrete support from spouses and mothers-in-law was limited, highlighting the need for awareness and adaptation strategies to address the effects of high heat exposure and maternal health in rural communities [4].

Previous research has found that temperature alone does not account for the whole-body heat balance and how the human body responds to conditions of a high heat thermal environment [5]. To better capture how different thermal environments influence the body response, heat metrics or indices are used in research in the maternal and perinatal health field [5]. Two such indices are the Wet Bulb Globe Temperature (WBGT) and Universal Thermal Climate Index (UTCI).

In this study, we used monthly aggregated data from 2017 until 2022 from Aga Khan University Hospital in Mombasa Kenya. We used a pooled time-series regression approach to compare the effects of heat exposure of temperature, WBGT and UTCI on different maternal and perinatal health outcomes.

## 2 Methods

Data were retrieved from the health surveillance system, the Aga Khan Hospital Surveillance Dashboard, on a monthly time scale for 2017 through 2022. On average, there were about 650 births in the hospital each year (Table 1). We evaluated the health outcomes of low Apgar score (score below 7 at 5 min), stillbirth (death of the baby after 28 weeks prior to delivery), preterm birth (birth before 37 weeks), long duration of stay in hospital (>5 days in hospital), assisted vaginal deliveries (avd), low birth weight (baby weighs <2500 g) and caesarean sections. A data sharing agreement was put in place between Aga Khan University and the University of Graz. Ethics agreement was approved by the University of Graz ethics committee. We follow the guidelines made available in the STROBE checklist for epidemiological studies, see the supplementary material [6].

**Table 1:** Descriptive statistics for birth outcomes from the Aga Khan University Hospital, Mombasa, brackets indicate the range in percentage of health outcomes.

Average Percent/ Years	2017	2018	2019	2020	2021	2022
Long Stay in Hospital	9.4 (3.6,17.4)	12.7 (2.2,29.4)	9.8 (2.5,30.8)	6.6 (0,13.7)	7.6 (2.8,15.8)	11.4 (4.5,18.9)
Low Apgar Score	2.1 (0,9.1)	2.3 (0,7.0)	1.7 (0,7.9)	2.0 (0,5.9)	3.4 (0,9.4)	1.3 (0,5.3)
Assisted Vaginal Delivery	10.7 (2.7,16.4)	9.4 (2.2,14.0)	8.5 (2.2,14.5)	9.4 3.0(17.2)	4.9 (0,12.5)	9.0 (2.4,15.8)
Caesarean Section	12.5 (0,31.0)	16.4 (7.7,38.4)	15.7 (6.6,28.3)	13.8 (4.5,26.0)	22.7 (5.459.2)	22.7 (9.2,44.4)
Low Birth Weight	6.5 (0,13.6)	8.5 (3.9,19.6)	6.8 (2.6,11.9)	5.8 (2.1,11.8)	8.1 (2.3,15.4)	8.1 (3.0,14.3)
Preterm Birth	4.5 (0,8.7)	7 (2,16.9)	8.8 (2.4,23.7)	6.3 (0,11.8)	6.3 (2.3,11.5)	5.9 (2.3,11.5)
Stillbirths	1.9 (0,8.1)	2.2 (0,5.6)	0.8 (0,4.8)	1.1 (0,7.8)	3.0 (0,7.7)	1.5 (0,5.3)
Live Births	98.2 (92.5,100)	97.9 (94.7,100)	99.2 (95.5,100)	98.9 (92.7,100)	97.2 (93.0,100)	98.5 (95,100)

### 2.1 Heat metrics

We made use of a state-of-the-art climate dataset ERA5, that provides global gridded data every 1 hour at a 0.25×0.25° grid scale [7]. We used three heat metrics: 2 m/air temperature, Universal Thermal Climate Index (UTCI) and Wet Bulb Globe Temperature (WBGT). WBGT is an international standard for heat stress in occupational settings. UTCI was created in the 2000s to address thermal stress in Europe [8]. We used the dataset known as ERA5-HEAT, which calculates UTCI using a polynomial approximation of a more complex model of the UTCI [9]. WBGT was created in the late 1950s to put in place protections for the US military against the hot environment [10]. We used a method created for ERA5 to calculate an accurate approximate of WBGT [11]. All three-heat metrics already have been used in research on maternal and perinatal health outcomes [5]. The climate and health data are on different scales, and this requires us to use geolocation and statistical downscaling. The health data for Mombasa Hospital in Kenya is geolocation point data. The approach taken was to interpolate using a weighted nearest neighbour method. In our approach, using the python library *xarray,* the point is selected with a tolerance of 0.2°; this matches the location to the nearest grid cell of the climate dataset ERA5 [12].

### 2.2 Distributed lag non-linear models

We made use of the dlnm R package, widely used in epidemiological analysis [13]. Quasi-Poisson distributed polynomial models were fitted to the dataset and we investigated the non-cumulative effect of heat exposure and appropriate cumulative exposure at lags up to 9 months prior. Outcomes are displayed as relative risk ratios of the rise in percentage of health outcomes observed in each month (i.e. number of babies born experiencing a health outcome in comparison to the total times by 100). These values are reported with a 95 % CI evaluating the increase from the median value of exposure which is known as the centroid to the 95th percentile. The median value was chosen as the comparative value because this is the value that newborns and mothers are most exposed to. Significant results are displayed in the manuscript, with all results available in the supplementary material.

## 3 Results

**Fig. 1:**
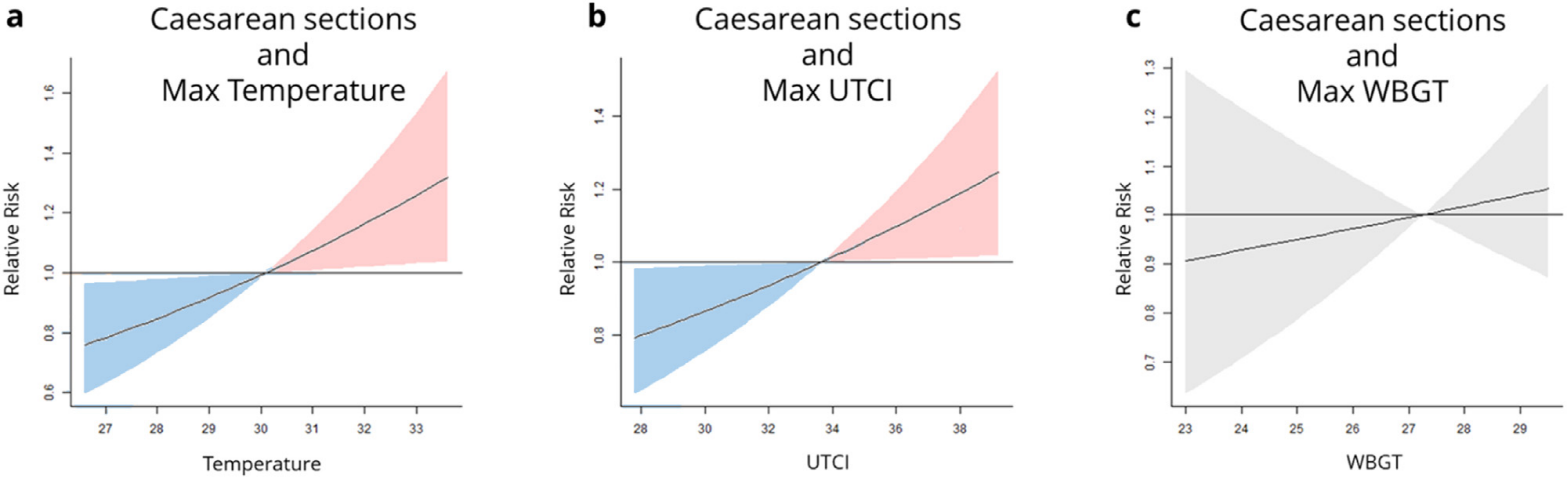
. The dose-response curves showing the influence of heat exposure on the health outcome of caesarean sections. A) is Maximum Temperature, and b) is Maximum UTCI, both show a positive response an increase in caesarean sections to heat exposure. C) is Maximum WBGT.

The odds of caesarean sections mode of delivery increased when only considering the month of birth at lag 0 for the heat metrics in the maximum of temperature and UTCI (Fig. 1). For example, the relative risk ratio at the 95th percentile in comparison to the median exposure was 1.21 (1.01,1.46) 95 % CI for maximum UTCI. There was a slightly higher relative risk of caesarean sections for maximum temperature where the relative risk ratio was 1.25 (1.03,1.53) 95 % CI. In all other heat metrics the relative risk was not significant. Considering cumulative exposure over the whole of pregnancy or over one trimester, is associated with no one month being significant (See Supplementary Material).

**Fig. 2:**
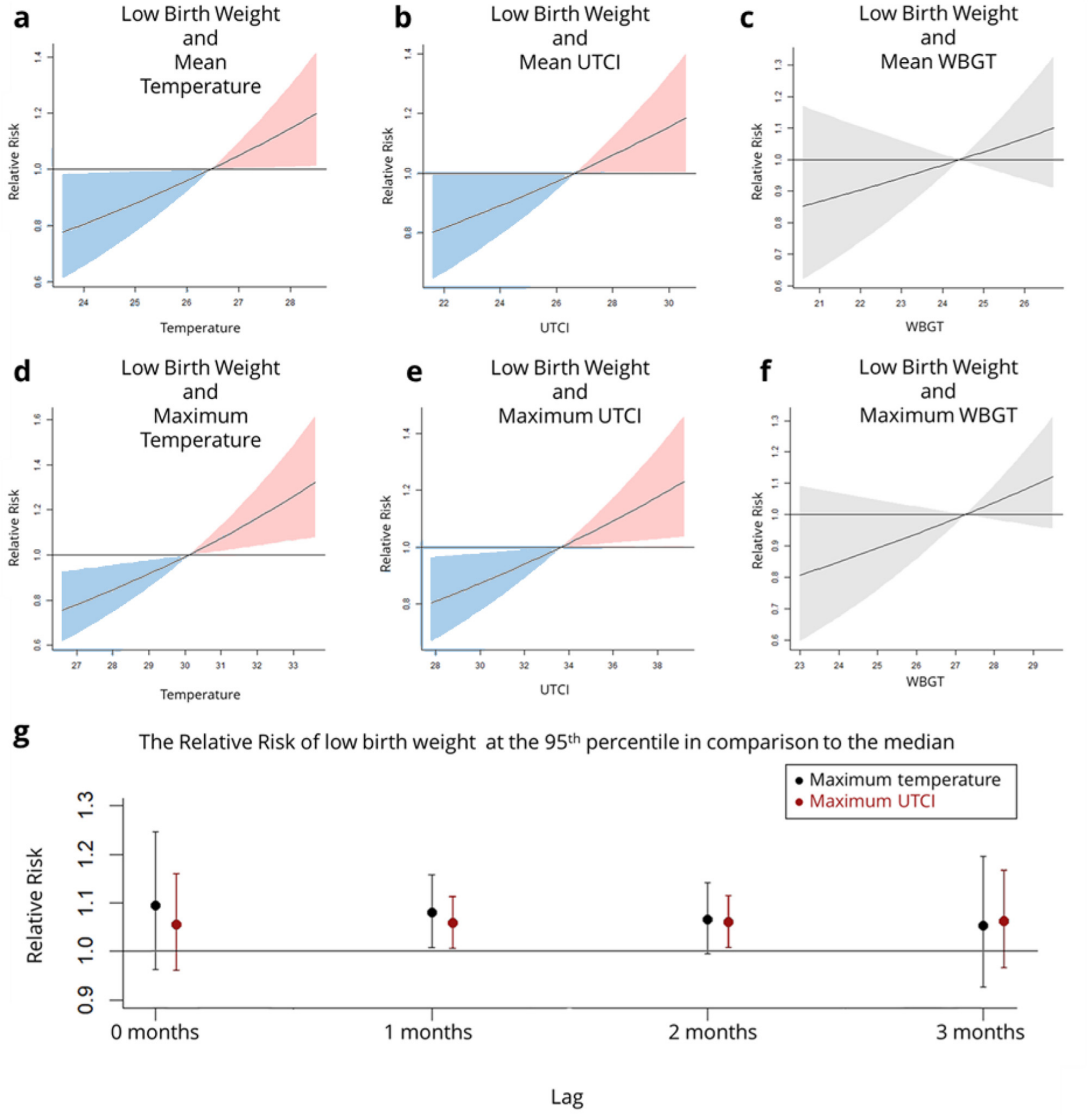
The dose-response curves showing the influence of heat exposure on the health outcome of Low Birth Weight (a-f). Where a) Mean Temperature, b) Mean UTCI, c) Mean WBGT and d) Maximum Temperature, e) Maximum UTCI and f) Maximum WBGT and the forest plot of the 95th percentile an indication of high heat exposure in comparison to median, most common exposure (g).

For the month of birth, lag 0, the odds of low birth weight (weights below 2500 g) increased for the heat metrics in the maximum and mean values of temperature and UTCI (Fig. 2). For low birth weight for maximum temperature, the relative risk ratio at the 95th percentile in comparison to the median exposure was 1.26 (1.06,1.51) 95 % CI and for mean temperature was 1.18 (1.01,1.37) 95 % CI. In comparison, for the maximum UTCI, the relative risk ratio was 1.20 (1.03,1.40) 95 % CI and for mean UTCI was 1.17 (1.00,1.35) 95 % CI. In all other heat metrics the relative risk is not significant.

When considering the cumulative exposure to heat over the whole of pregnancy, there is no significant rise in incidence of low birth weight. However, if only the last trimester is considered, exposure in the 2 months prior, both 2 months and 1 month before birth is significantly associated with an increase in the relative risk of a rise in the percentages of low-birth-weight babies (Fig. 2). The relative risk ratio for 2 months lag for Maximum UTCI at the 95th percentile in comparison to the median exposure was 1.07 (1.01,1.14) 95 % CI. The relative risk ratio for 1 month lag for Maximum Temperature was 1.08 (1.01,1.16) 95 % CI and for Maximum UTCI was 1.07 (1.01,1.13) 95 % CI. Results for other outcomes were not significant and can be seen in the supplementary material.

## 4 Discussion

We found that whilst no outcome is significantly influenced by WBGT, low birth weight and caesarean sections were significantly influenced by non-cumulative exposure to maximum UTCI and temperature. In addition, we found differences in significance depending on the minimum, mean or maximum values used as an indication of extreme heat exposure. In contrast, most literature to date has suggested that there was no difference in the observed response across different heat metrics in comparison to temperature and adverse birth and maternal health outcomes [3,5]. Literature also demonstrated a reluctancy to use heat metrics in epidemiological modelling and comparing outputs of heat metrics and temperature as like for like values. It is apparent that monthly mean of the daily maximum, temperature, UTCI and WBGT values have different distributions (see Supplementary Material) in comparison to percentage of low birth weights and percentage of caesarean sections. This accounts for the difference observed in the health outcome responses to the different heat metrics and the level of significance observed. In addition, it gives credence that heat metrics and temperature are not like for like values [5].

With climate change it is known that heat exposure of populations is increasing and that potential tipping points exist, beyond which human biological survivability in most exposed regions might be seriously hampered [14,15]. Our study provides evidence towards such potential tipping points, by showing an example of a heat threshold at which maternal women and neonates experience an increase in the number of low-birth-weight babies and number of caesarean sections, showing a significant positive association above the median exposure of around 26 °C in the mean UTCI. For the original warning thresholds for this heat metric, this is equivalent to moderate heat stress [9]. This provides an additional argument supporting efforts in reducing anthropogenic greenhouse gas emissions and lowering the global temperature rise [1,14]. Limiting the increase in heat extremes will positively benefit the most vulnerable groups by reducing a rise in heat exposure [1,14]. However, more research is needed to further assess such tipping points and heat thresholds for health risks for heat vulnerable groups.

There is a gap in literature for health responses in neonates and maternal women and WBGT with very few studies assessing these groups [5]. It could be that the lack of significance of any outcome to exposure to WBGT was due to the non-dynamical response of the index to wind speed, which has been shown in some studies to lead to inaccuracies in predicting heat strain [16,17]. More research is needed in different environments for WBGT and diverse study populations.

We showed a significant rise in the odds of caesarean sections with exposure to heat. Considering the real-world context, it is most likely that caesarean sections in Kenya are predominantly emergency procedures as 72 % of caesarean sections in sub-Saharan Africa are emergency procedures [18]. We can therefore hypothesize that instantaneous exposure to heat in the month of birth would be more impactful as the decision is taken in a short period. There is a lack of precedence in literature aswe could find only one prior study regarding emergency caesarean sections for preterm births in the US [19]. In the past, research has found that in Central Kenya, caesarean sections were associated with 48 % of maternal mortalities due to haemorrhage [20]. Future research could therefore consider if there is a rise in maternal mortality and amount of blood loss during labour with exposure to heat in Kenya.

Our results were consistent with some other studies that use the same heat metrics and birth outcomes for other regions. For example, a study on stillbirth in Ghana using WBGT as the heat exposure metric also found no significant trends [21]. In contrast, our results were at odds with a study on stillbirth in Western Australia that found a significant positive association of an increase in stillbirths with higher values of UTCI; this could be explained by the differences in methodological approaches [22]. For example, the study in Western Australia used a case-crossover design where individuals acted as their own controls for exposure, whereas here a pooled time series analysis took place, only considering the influence of monthly exposure to heat.

Our results were consistent with other studies on low birth weight. In a study in Southern China it was reported for every 1 % increase in the UTCI there was a 22.4-gram (SE 9.4) reduction in birth weight [23]. Another research team using the heat metric apparent temperature found exposure to extreme heat in the last trimester of pregnancy was associated with a rise in low-birth-weight babies [24].

In conclusion, our research provides insights on the relationship between heat metrics and adverse birth and maternal health outcomes in Mombasa, Kenya. While previous literature often treated heat metrics and temperature interchangeably, our findings suggest distinct effects on health outcomes. Specifically, we observed significant influences of non-cumulative exposure to maximum UTCI and temperature for the health outcomes of Low Birth Weight and caesarean sections whilst no significance for WBGT, challenging the notion of uniform health responses across different heat metrics. These findings highlight the importance of considering various heat metrics independently rather than assuming they are the same as temperature. In addition, we provide evidence towards heat tipping points, demonstrating the need to concentrate efforts to reduce global warming and the associated increase in heat extremes. Our study highlights a critical research gap in understanding the effects of heat on neonatal and maternal health, particularly concerning heat metrics, and further research in diverse study environments and populations is warranted. Our results for emergency caesarean sections in Kenya suggest more immediate heat exposure may have a greater impact on outcomes and that more research on this type of delivery is needed. In summary, our study provides valuable insights into the relationship between heat exposure and maternal and perinatal health outcomes in Kenya, offering a foundation for future research and policy interventions aimed at understanding the adverse effects of heat on vulnerable populations.

## 5 Limitations

The Mombasa Hospital Dataset is a small dataset and we may see significance in other outcomes with a larger dataset [25] . There are further limitations that this data is only representative of a single tertiary hospital, not taking into account other types of healthcare facilities or births in other settings. It is widely acknowledged that the socio-economic status of mothers has a significant influence in addition to heat exposure in influencing adverse birth outcomes [5] . Nevertheless, no socio-economic data was recorded in this dataset and consequently it was not possible to explore the influence of socio-economic characteristics on vulnerability.

## Patient and public involvement

Patients and the Public were not involved in the study.

## Role of the funding source

The funders had no role in the design of the study or the subsequent results.

## Data sharing statement

The climate data is available from: https://zenodo.org/records/8,021,197, https://doi.org/10.24381/cds.553b7518 and https://10.24381/cds.adbb2d47. The data analysis protocol for the HIGH Horizons project covering this study is available at: https://doi.org/10.5281/zenodo.10947995, code is available on request from the corresponding author.

## Funding

This research was funded by the European Union Horizon programme as part of the HIGH Horizons project under grant agreement number 101057843. LSHTM is funded by UKRI Innovate UK reference number 10038478. Professor Jackson is also funded by the Takeda Foundation. Open Acess funding is provided by the University of Graz.

## CRediT authorship contribution statement

**Chloe Brimicombe:** Writing – review & editing, Writing – original draft, Visualization, Resources, Methodology, Investigation, Formal analysis, Conceptualization. **Debra Jackson:** Writing – review & editing, Resources, Methodology, Conceptualization. **Aquinius Mungatia:** Writing – review & editing, Data curation. **Zeenat Sulaiman:** Writing – review & editing, Funding acquisition. **Tobias Monthaler:** Writing – review & editing, Data curation. **Katharina Wieser:** Writing – review & editing, Visualization, Methodology. **Ilona M Otto:** Writing – review & editing, Supervision, Resources, Methodology, Funding acquisition, Conceptualization.

## Declaration of competing interest

The authors report there are no competing interests to declare.

## Appendix Supplementary materials

Image, application 1

Image, application 2
