# Supplementary material for: The influence of heat exposure on birth and neonatal outcomes in Mombasa, Kenya: A pooled time series analysis
Source: J Clim Chang Health. 2025 Jan 15;22:100409. doi: 10.1016/j.joclim.2024.100409 (PMC12851306; doi:10.1016/j.joclim.2024.100409)
Supplement: Supplementary file 1 [file mmc1.docx]

STROBE Statement—checklist of items that should be included in reports of observational studies

|  | Item No. | Recommendation | Section No. | Relevant text from manuscript |
| --- | --- | --- | --- | --- |
| **Title and abstract** | 1 | (*a*) Indicate the study’s design with a commonly used term in the title or the abstract | Title | The influence of heat exposure on birth and neonatal outcomes in Mombasa, Kenya: a pooled time series analysis |
|  |  | (*b*) Provide in the abstract an informative and balanced summary of what was done and what was found | Abstract | We observed an increased odds of caesarean sections with heat exposure at lag 0 indicated by maximum daily Universal Thermal Climate Index (UTCI) between the 50th and the 95th percentile (relative risk 1.21 (1.01,1.46, 95%CI)) and maximum daily temperature (1.25 (1.53, 1.03)). There were increased odds of Low-Birth-Weight Births for lag 0 mean and maximum UTCI. We find no significant responses for Wet Bulb Globe Temperature (WBGT). |
| Introduction | | | |  |
| Background/rationale | 2 | Explain the scientific background and rationale for the investigation being reported | 1 | i.e. Exposure to extreme heat can lead to adverse birth and maternal health outcomes [3]. In the context of Kenya, one study discussed the influence of heat on maternal health and wellbeing in rural Kilifi in coastal Kenya |
| Objectives | 3 | State specific objectives, including any prespecified hypotheses | 1 | We used a pooled time-series regression approach to compare the effects of heat exposure of temperature, WBGT and UTCI on different maternal and perinatal health outcomes. |
| Methods | | | |  |
| Study design | 4 | Present key elements of study design early in the paper | 2 | In this study, we used monthly aggregated data from 2017 until 2022 from Aga Khan University Hospital in Mombasa Kenya. We used a pooled time-series regression approach to compare the effects of heat exposure of temperature, WBGT and UTCI on different maternal and perinatal health outcomes. |
| Setting | 5 | Describe the setting, locations, and relevant dates, including periods of recruitment, exposure, follow-up, and data collection | 2 | Data were retrieved from the health surveillance system the Aga Khan Hospital Surveillance Dashboard on a monthly time scale for 2017 through 2022. |
| Participants | 6 | (*a*) *Cohort study*—Give the eligibility criteria, and the sources and methods of selection of participants. Describe methods of follow-up  *Case-control study*—Give the eligibility criteria, and the sources and methods of case ascertainment and control selection. Give the rationale for the choice of cases and controls  *Cross-sectional study*—Give the eligibility criteria, and the sources and methods of selection of participants | 2 | Data were retrieved from the health surveillance system the Aga Khan Hospital Surveillance Dashboard on a monthly time scale for 2017 through 2022. |
|  |  | (*b*) *Cohort study*—For matched studies, give matching criteria and number of exposed and unexposed  *Case-control study*—For matched studies, give matching criteria and the number of controls per case | 2 | On average, there were about 650 births in the hospital each year (Table 1). |
| Variables | 7 | Clearly define all outcomes, exposures, predictors, potential confounders, and effect modifiers. Give diagnostic criteria, if applicable | 2 | We evaluated the health outcomes of low Apgar score (score below 7 at 5 minutes), stillbirth (death of the baby after 28 weeks prior to delivery), preterm birth (birth before 37 weeks), long duration of stay in hospital (more than 5 days in hospital), assisted vaginal deliveries (AVD), low birth weight (baby weighs less than 2500 grams) and caesarean sections. |
| Data sources/ measurement | 8* | For each variable of interest, give sources of data and details of methods of assessment (measurement). Describe comparability of assessment methods if there is more than one group | 2 | Data were retrieved from the health surveillance system the Aga Khan Hospital Surveillance Dashboard on a monthly time scale for 2017 through 2022. |
| Bias | 9 | Describe any efforts to address potential sources of bias | 5 | The Mombasa Hospital Dataset is a small dataset and so it may be that we would see significance in other outcomes with a larger dataset [24] . There are further limitations that this data is only representative of a single tertiary hospital, not taking into account over types of healthcare facility or births in other settings. |
| Study size | 10 | Explain how the study size was arrived at | 2 | Data were retrieved from the health surveillance system the Aga Khan Hospital Surveillance Dashboard on a monthly time scale for 2017 through 2022. |

Continued on next page

| Quantitative variables | 11 | Explain how quantitative variables were handled in the analyses. If applicable, describe which groupings were chosen and why | 2 | All three-heat metrics already have been used in research on maternal and perinatal health outcomes [5].  Outcomes are displayed as relative risk ratios of the rise in percentage of health outcomes observed in each month (i.e. number of babies born experiencing a health outcome in comparison to the total times by 100). |
| --- | --- | --- | --- | --- |
| Statistical methods | 12 | (*a*) Describe all statistical methods, including those used to control for confounding | 5 | It is widely acknowledged that socio-economic status of mothers has a significant influence in addition to heat exposure in influencing adverse birth outcomes [5] . Nevertheless, no socio-economic data was recorded in this dataset and consequently it was not possible to explore the influence of socio-economic characteristics on vulnerability. |
|  |  | (*b*) Describe any methods used to examine subgroups and interactions | 5 | It is widely acknowledged that socio-economic status of mothers has a significant influence in addition to heat exposure in influencing adverse birth outcomes [5] . Nevertheless, no socio-economic data was recorded in this dataset and consequently it was not possible to explore the influence of socio-economic characteristics on vulnerability. |
|  |  | (*c*) Explain how missing data were addressed |  | n/a |
|  |  | (*d*) *Cohort study*—If applicable, explain how loss to follow-up was addressed  *Case-control study*—If applicable, explain how matching of cases and controls was addressed  *Cross-sectional study*—If applicable, describe analytical methods taking account of sampling strategy | 2 | We made use of the dlnm R package, widely used in epidemiological analysis [12]. Quasi-Poisson distributed polynomial models were fitted to the dataset and we investigated the non-cumulative effect of heat exposure and appropriate cumulative exposure at lags up to 9 months prior. |
|  |  | (*e*) Describe any sensitivity analyses | 5 | It is widely acknowledged that socio-economic status of mothers has a significant influence in addition to heat exposure in influencing adverse birth outcomes [5] . Nevertheless, no socio-economic data was recorded in this dataset and consequently it was not possible to explore the influence of socio-economic characteristics on vulnerability. |
| Results | | | | |
| Participants | 13* | (a) Report numbers of individuals at each stage of study—eg numbers potentially eligible, examined for eligibility, confirmed eligible, included in the study, completing follow-up, and analysed | 2 | On average, there were about 650 births in the hospital each year (Table 1). |
|  |  | (b) Give reasons for non-participation at each stage |  | n/a |
|  |  | (c) Consider use of a flow diagram |  | n/a |
| Descriptive data | 14* | (a) Give characteristics of study participants (eg demographic, clinical, social) and information on exposures and potential confounders | 5 | It is widely acknowledged that socio-economic status of mothers has a significant influence in addition to heat exposure in influencing adverse birth outcomes [5] . Nevertheless, no socio-economic data was recorded in this dataset and consequently it was not possible to explore the influence of socio-economic characteristics on vulnerability. |
|  |  | (b) Indicate number of participants with missing data for each variable of interest |  | n/a |
|  |  | (c) *Cohort study*—Summarise follow-up time (eg, average and total amount) |  | n/a |
| Outcome data | 15* | *Cohort study*—Report numbers of outcome events or summary measures over time |  | *n/a* |
|  |  | *Case-control study—*Report numbers in each exposure category, or summary measures of exposure |  | *n/a* |
|  |  | *Cross-sectional study—*Report numbers of outcome events or summary measures | *2* | On average, there were about 650 births in the hospital each year (Table 1). |
| Main results | 16 | (*a*) Give unadjusted estimates and, if applicable, confounder-adjusted estimates and their precision (eg, 95% confidence interval). Make clear which confounders were adjusted for and why they were included | 2 | Outcomes are displayed as relative risk ratios of the rise in percentage of health outcomes observed in each month (i.e. number of babies born experiencing a health outcome in comparison to the total times by 100). These values are reported with a 95% CI evaluating the increase from the median value of exposure which is known as the centroid. to the 95th percentile. Median value is chosen as the comparative value because this is the value that newborns and mothers are most exposed to. Significant results are displayed in the manuscript, with all results available in the supplementary material. |
|  |  | (*b*) Report category boundaries when continuous variables were categorized |  | n/a |
|  |  | (*c*) If relevant, consider translating estimates of relative risk into absolute risk for a meaningful time period |  | n/a |

Continued on next page

| Other analyses | 17 | Report other analyses done—eg analyses of subgroups and interactions, and sensitivity analyses | 2 | Significant results are displayed in the manuscript, with all results available in the supplementary material. |
| --- | --- | --- | --- | --- |
| Discussion | | | | |
| Key results | 18 | Summarise key results with reference to study objectives | 4 | We found that whilst no outcome is significantly influenced by WBGT; Low Birth Weight and Caesarean Sections were significantly influenced by non-cumulative exposure to maximum UTCI and temperature. In addition, we found differences in significance depending on the minimum, mean or maximum values used as an indication of extreme heat exposure. In contrast, most literature to date suggested that there was no difference in the observed response across different heat metrics in comparison to temperature and adverse birth and maternal health outcomes [3,5]. |
| Limitations | 19 | Discuss limitations of the study, taking into account sources of potential bias or imprecision. Discuss both direction and magnitude of any potential bias | 5 | The Mombasa Hospital Dataset is a small dataset and so it may be that we would see significance in other outcomes with a larger dataset [24] . There are further limitations that this data is only representative of a single tertiary hospital, not taking into account over types of healthcare facility or births in other settings. It is widely acknowledged that socio-economic status of mothers has a significant influence in addition to heat exposure in influencing adverse birth outcomes [5] . Nevertheless, no socio-economic data was recorded in this dataset and consequently it was not possible to explore the influence of socio-economic characteristics on vulnerability. |
| Interpretation | 20 | Give a cautious overall interpretation of results considering objectives, limitations, multiplicity of analyses, results from similar studies, and other relevant evidence | 4 | In conclusion, our research shone light on the relationship between heat metrics and adverse birth and maternal health outcomes in Mombasa, Kenya. While previous literature often treated heat metrics and temperature interchangeably, our findings suggest distinct effects on health outcomes. Specifically, we observed significant influences of non-cumulative exposure to maximum UTCI and temperature for the health outcomes of Low Birth Weight and Caesarean Sections whilst no significance for WBGT challenging the notion of uniform health responses across different heat metrics. |
| Generalisability | 21 | Discuss the generalisability (external validity) of the study results | 4 | In addition, this highlights the importance of considering various heat metrics independently rather than assuming they are the same as temperature. In addition, we provide evidence towards heat tipping points, demonstrating the need to concentrate efforts to reduce global warming and the associated increase in heat extremes. In addition, our study highlights a critical research gap in understanding the effects of heat on neonatal and maternal health, particularly concerning heat metrics, further research in diverse study environments and populations is warranted. |
| Other information | |  | | |
| Funding | 22 | Give the source of funding and the role of the funders for the present study and, if applicable, for the original study on which the present article is based |  | This research was funded by the European Union’s Horizons programme as part of the HIGH Horizons project under grant agreement number 101057843. LSHTM is funded by UKRI Innovate UK reference number 10038478. Professor Jackson is also funded by the Takeda Foundation. |

*Give information separately for cases and controls in case-control studies and, if applicable, for exposed and unexposed groups in cohort and cross-sectional studies.

**Note:** An Explanation and Elaboration article discusses each checklist item and gives methodological background and published examples of transparent reporting. The STROBE checklist is best used in conjunction with this article (freely available on the Web sites of PLoS Medicine at http://www.plosmedicine.org/, Annals of Internal Medicine at http://www.annals.org/, and Epidemiology at http://www.epidem.com/). Information on the STROBE Initiative is available at www.strobe-statement.org.
