# Supplementary material for: The influence of heat exposure on birth and neonatal outcomes in Mombasa, Kenya: A pooled time series analysis
Source: J Clim Chang Health. 2025 Jan 15;22:100409. doi: 10.1016/j.joclim.2024.100409 (PMC12851306; doi:10.1016/j.joclim.2024.100409)
Supplement: Supplementary file 2 [file mmc2.docx]

**Supplementary Materials for the influence of heat exposure on birth and neonatal outcomes in Mombasa, Kenya: a pooled time series analysis**


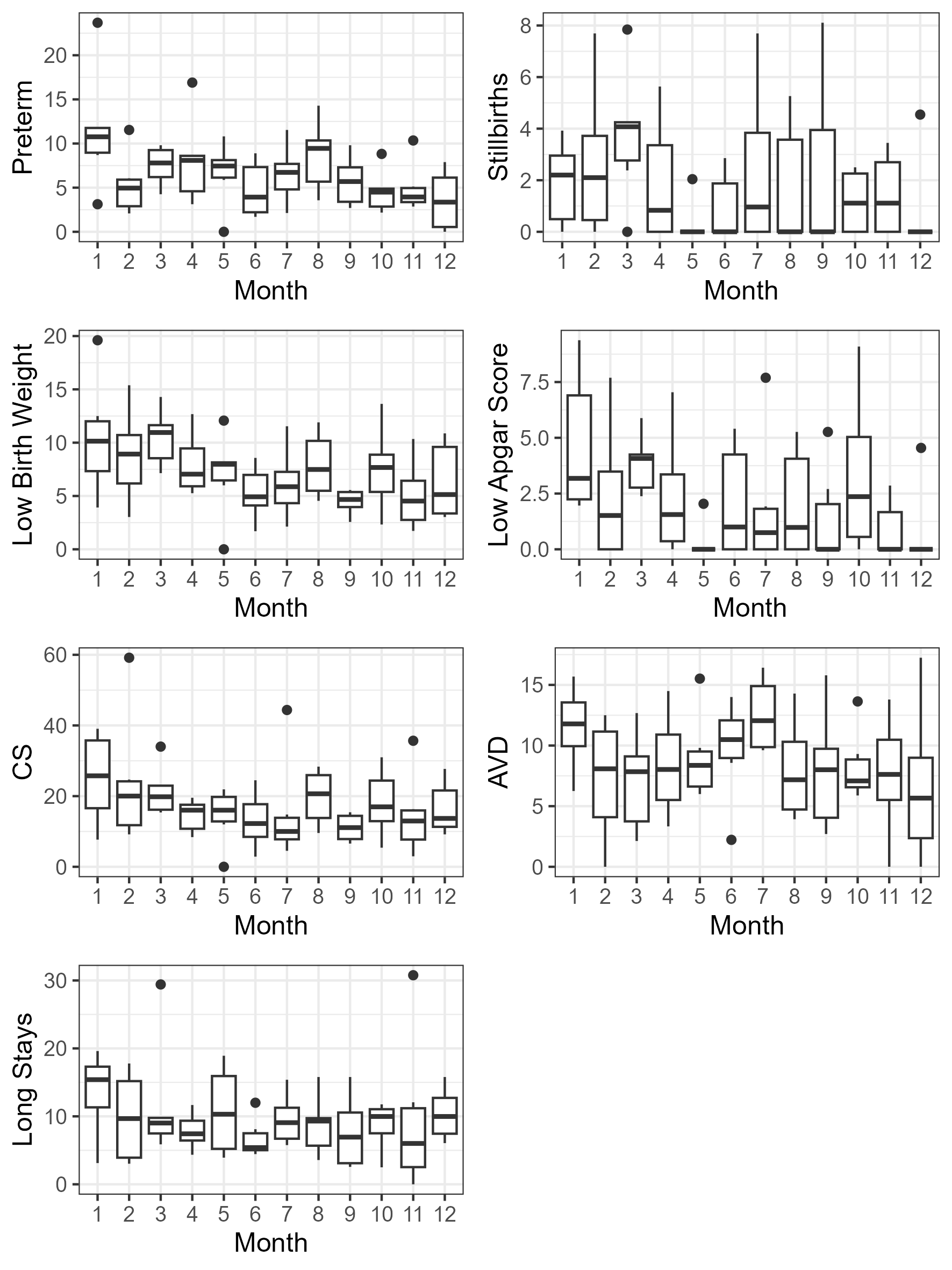


Figure S1: Seasonal distribution by month over 2017 to 2022 of percentage occurrence of health outcomes. Preterm, Stillbirths, Caesarean Section Delivery, Long Stays in Hospital, Stillbirths, Low Apgar score and Assisted Vaginal Delivery (AVD).


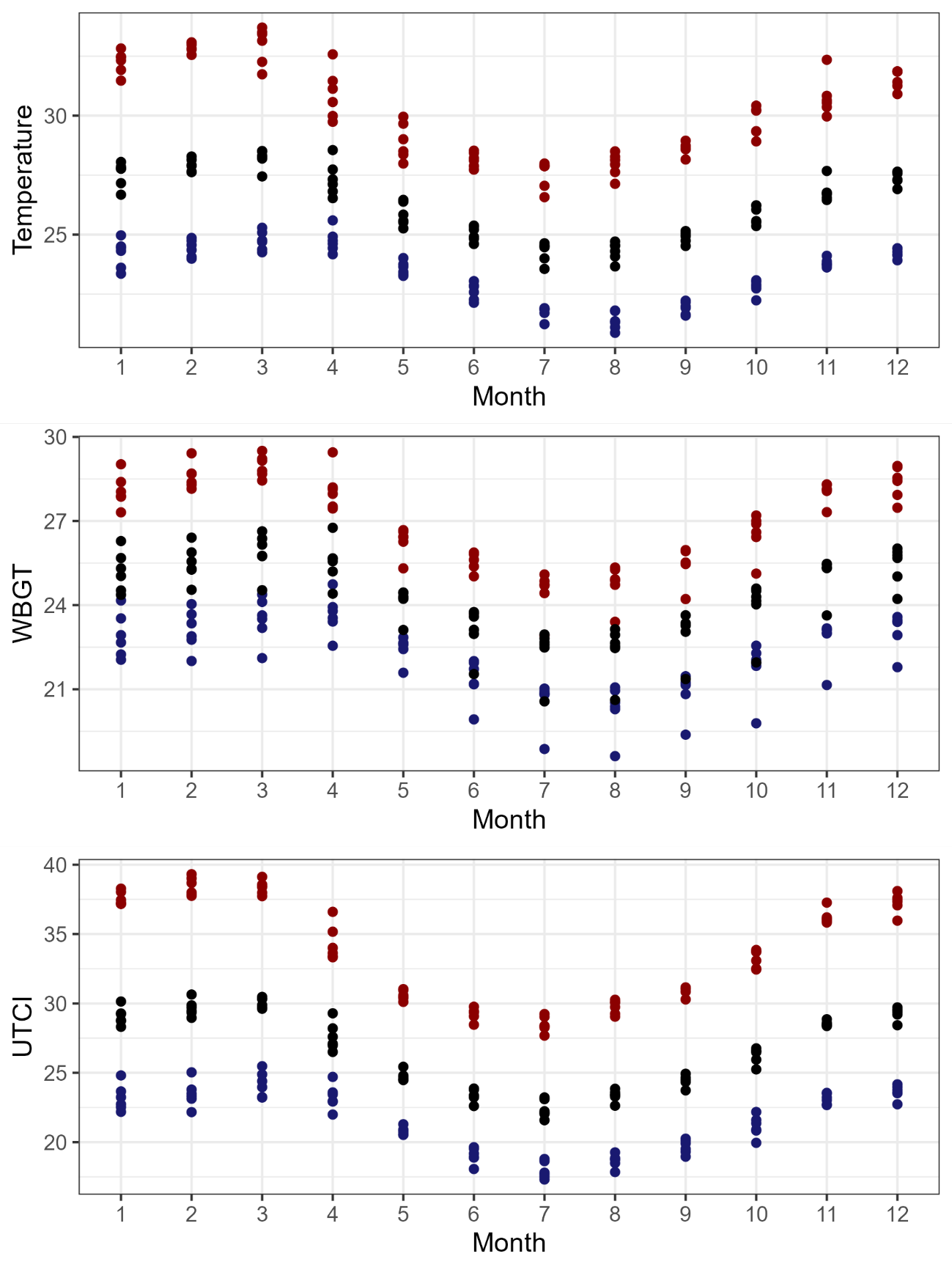


Figure S2: Season distribution of heat metrics temperature, wbgt and utci by month for 2017 to 2022. Red is maximum, black is mean and blue is minimum values.


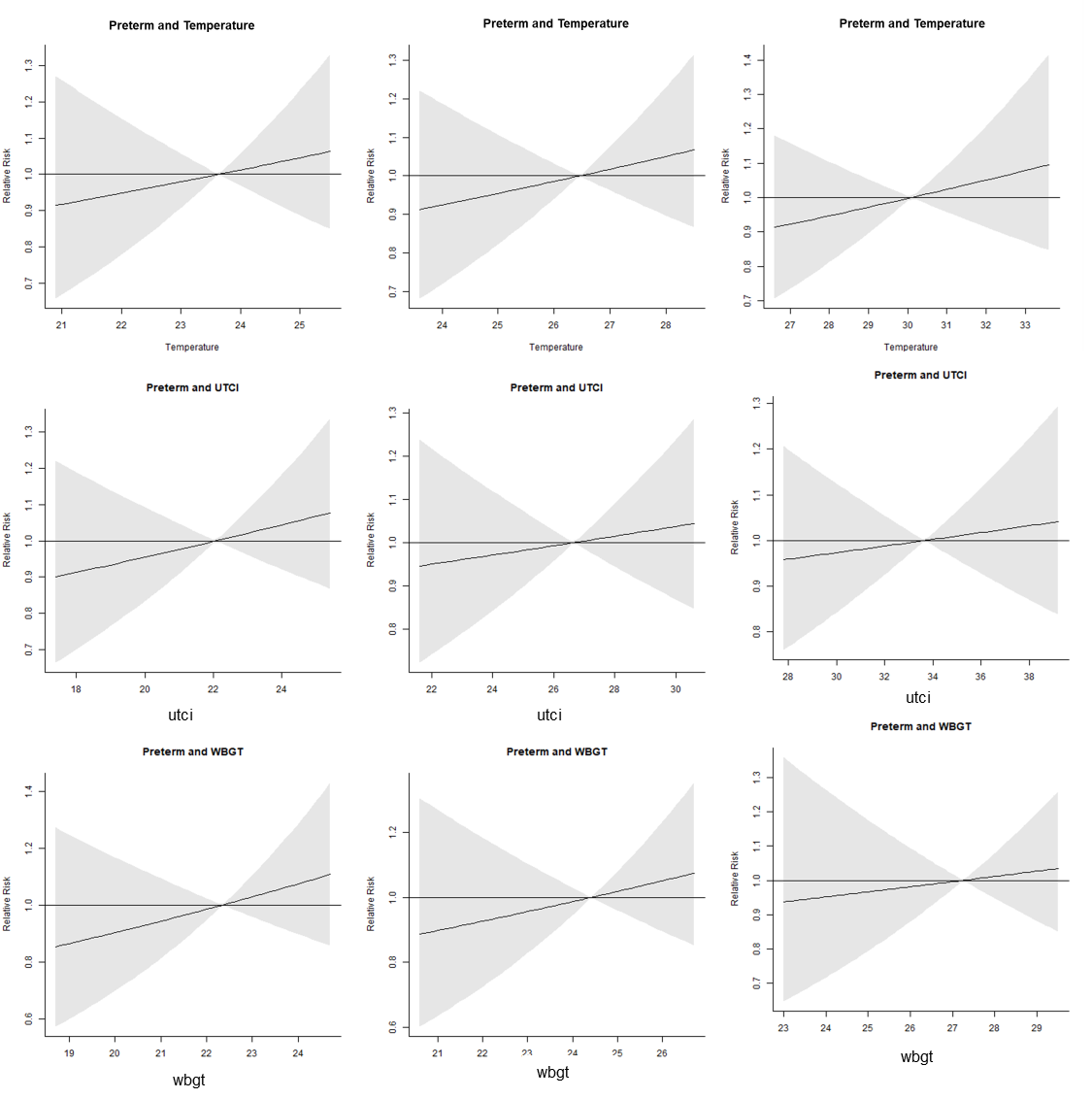


Figure S3: Relative Risk of higher percentages of preterm birth and exposure to heat as indicated by three heat metrics (R to L) monthly mean of daily minimum, mean and maximum temperature (top) utci (middle) and wbgt (bottom).


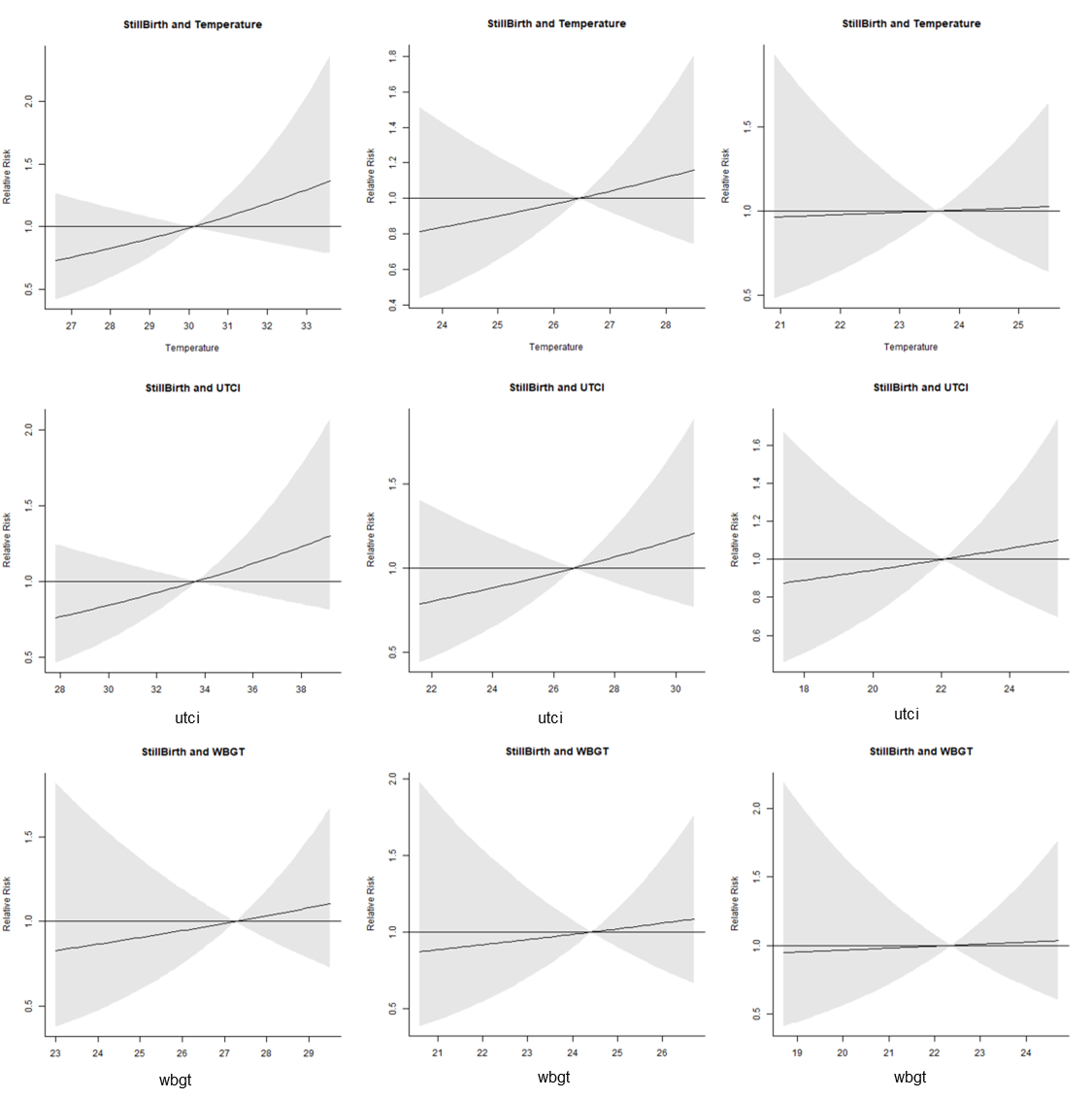


Figure S4: Relative Risk of higher percentages of stillbirth and exposure to heat as indicated by three heat metrics (R to L) monthly mean of daily minimum, mean and maximum temperature (top) utci (middle) and wbgt (bottom).


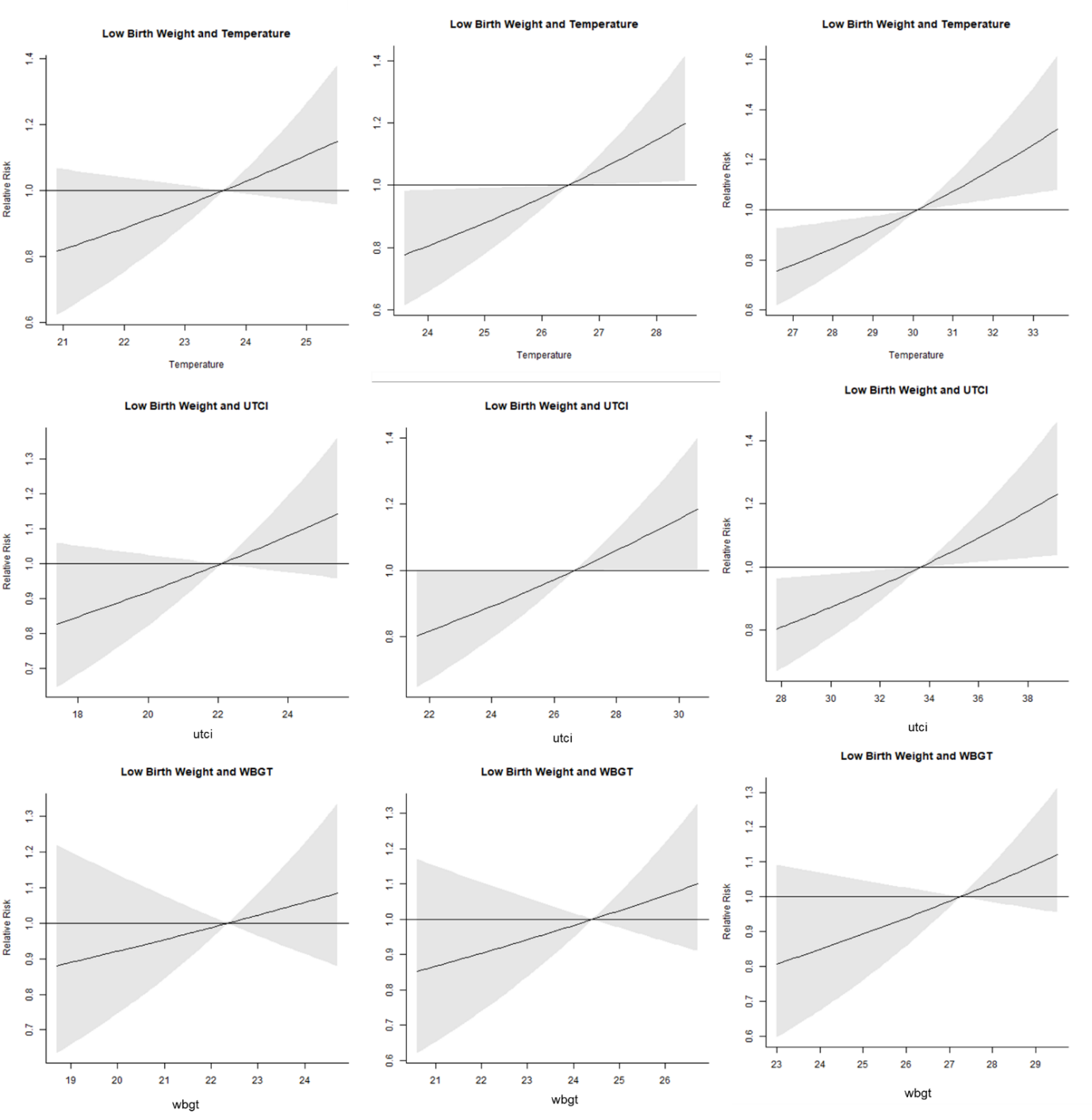


Figure S5: Relative Risk of higher percentages of low birth weights and exposure to heat as indicated by three heat metrics (R to L) monthly mean of daily minimum, mean and maximum temperature (top) utci (middle) and wbgt (bottom).


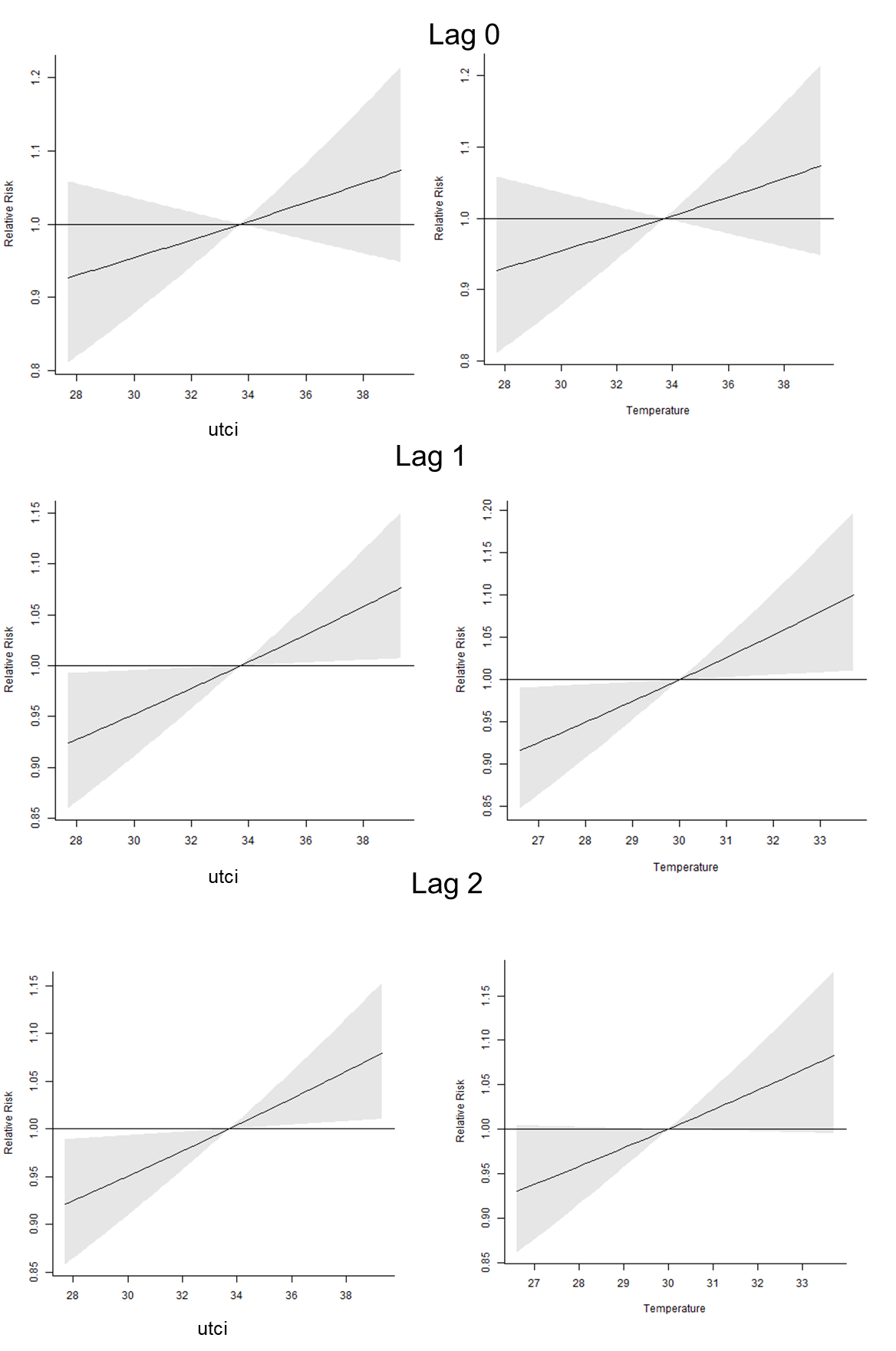


Figure S6: The relative risk of a rise in the percentage of low-birth-weight births with exposure to (L to R) maximum utci and temperature values. Lag 0 (Top), Lag 1(middle), Lag 2 (bottom).


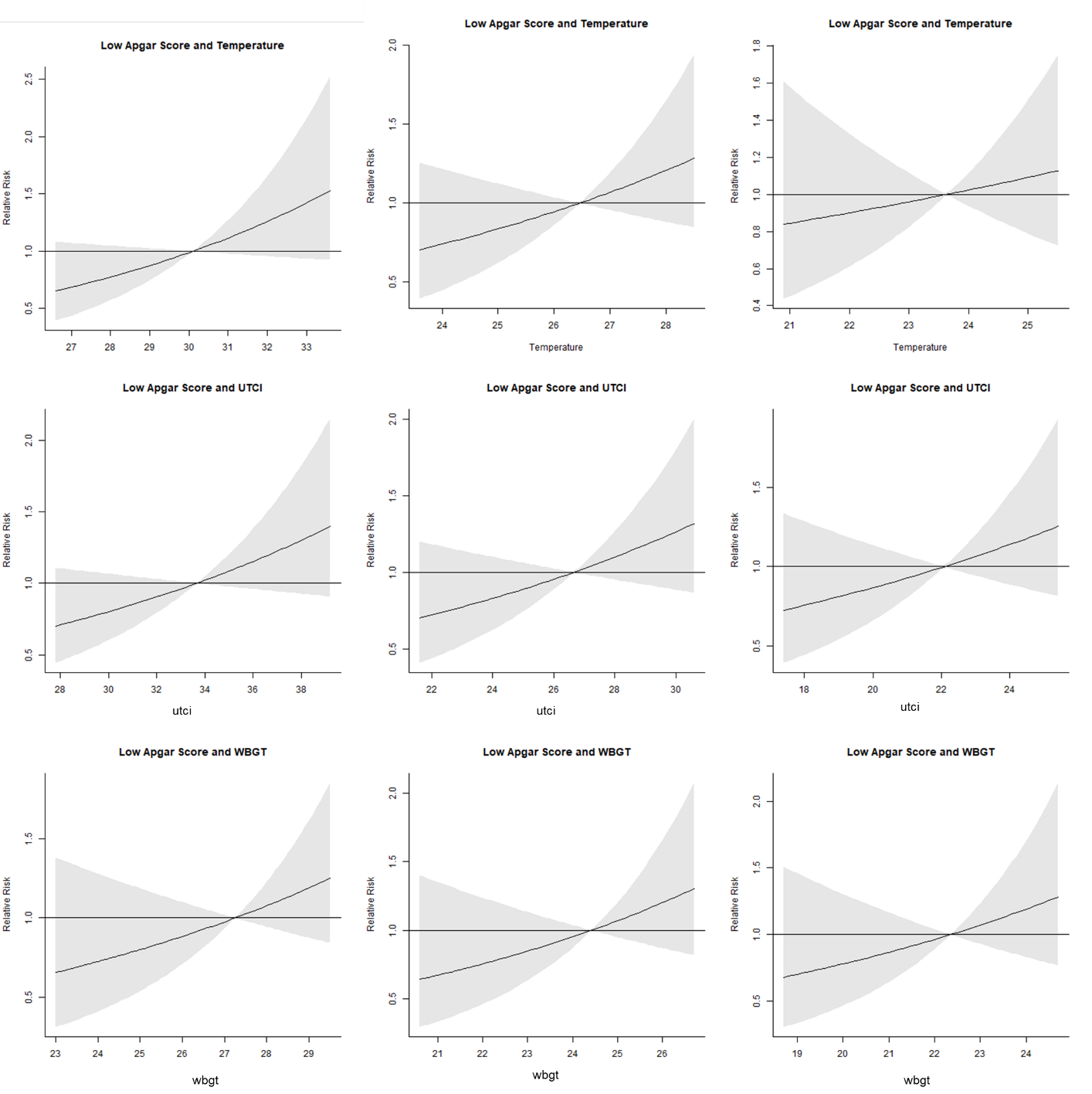


Figure S7 Relative Risk of higher percentages of low Apgar score and exposure to heat as indicated by three heat metrics (R to L) monthly mean of daily minimum, mean and maximum temperature (top) utci (middle) and wbgt (bottom).


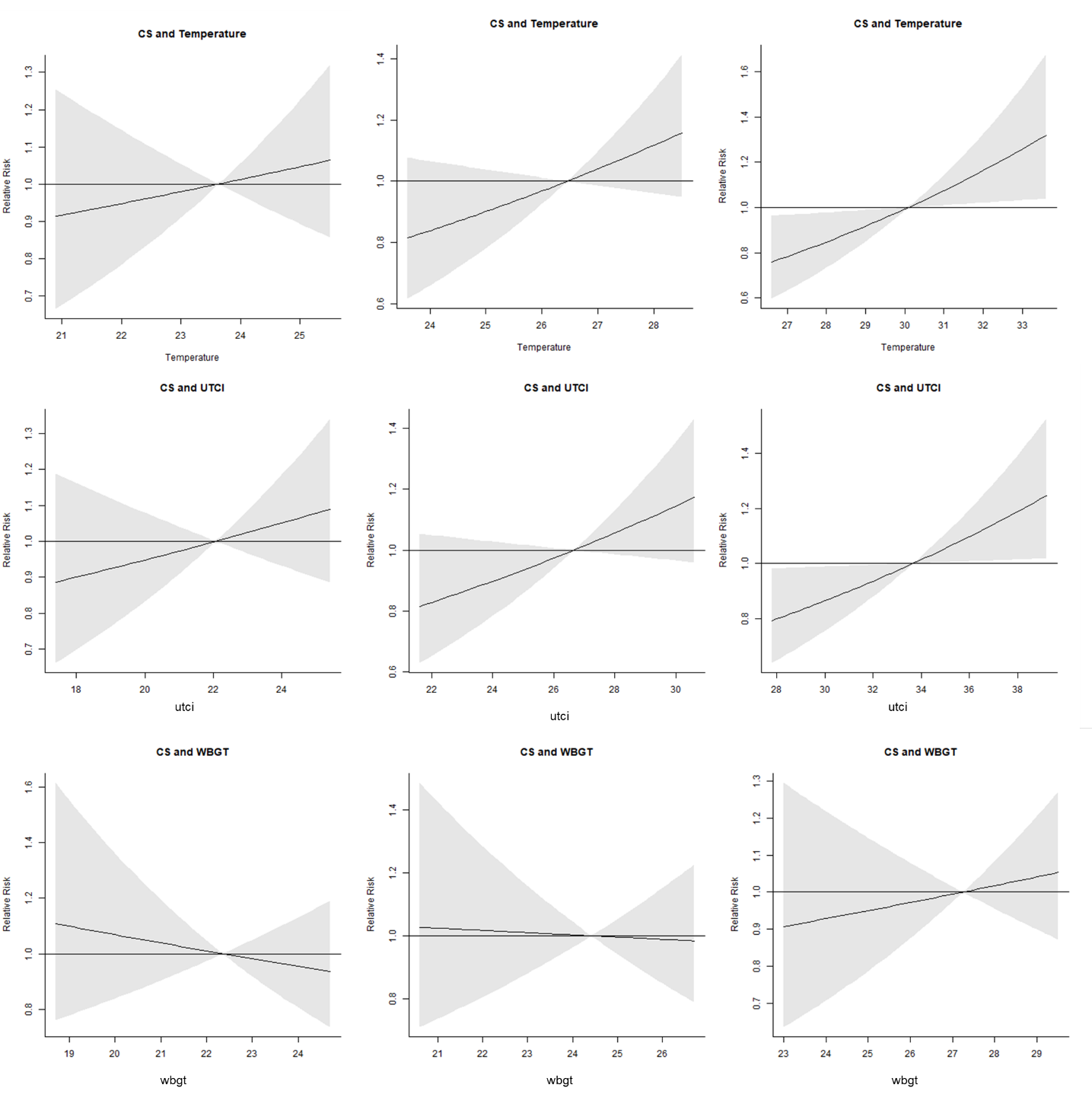


Figure S8: Relative Risk of higher percentages of Caesarean Sections (CS) and exposure to heat as indicated by three heat metrics (R to L) monthly mean of daily minimum, mean and maximum temperature (top) utci (middle) and wbgt (bottom).


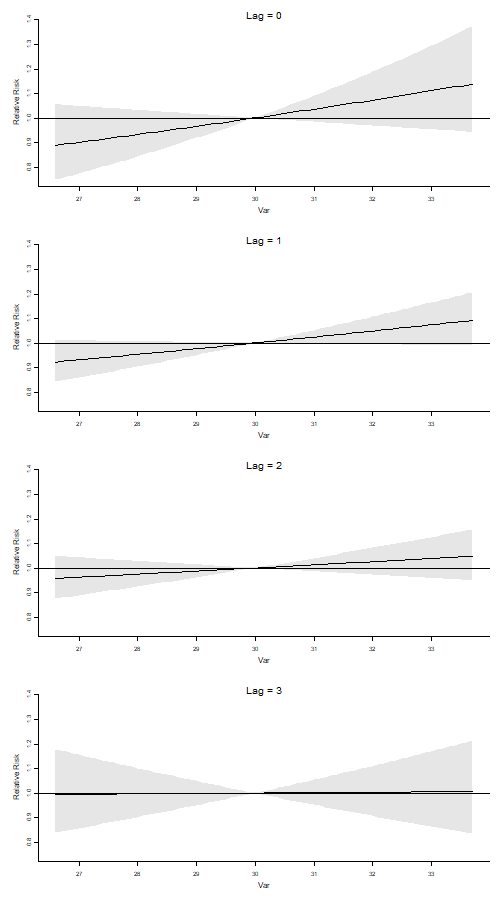


Figure S9 Relative risk of a rise in percentage of Caesarean Sections due to cumulative exposure to maximum temperature values over the last trimester of pregnancy.


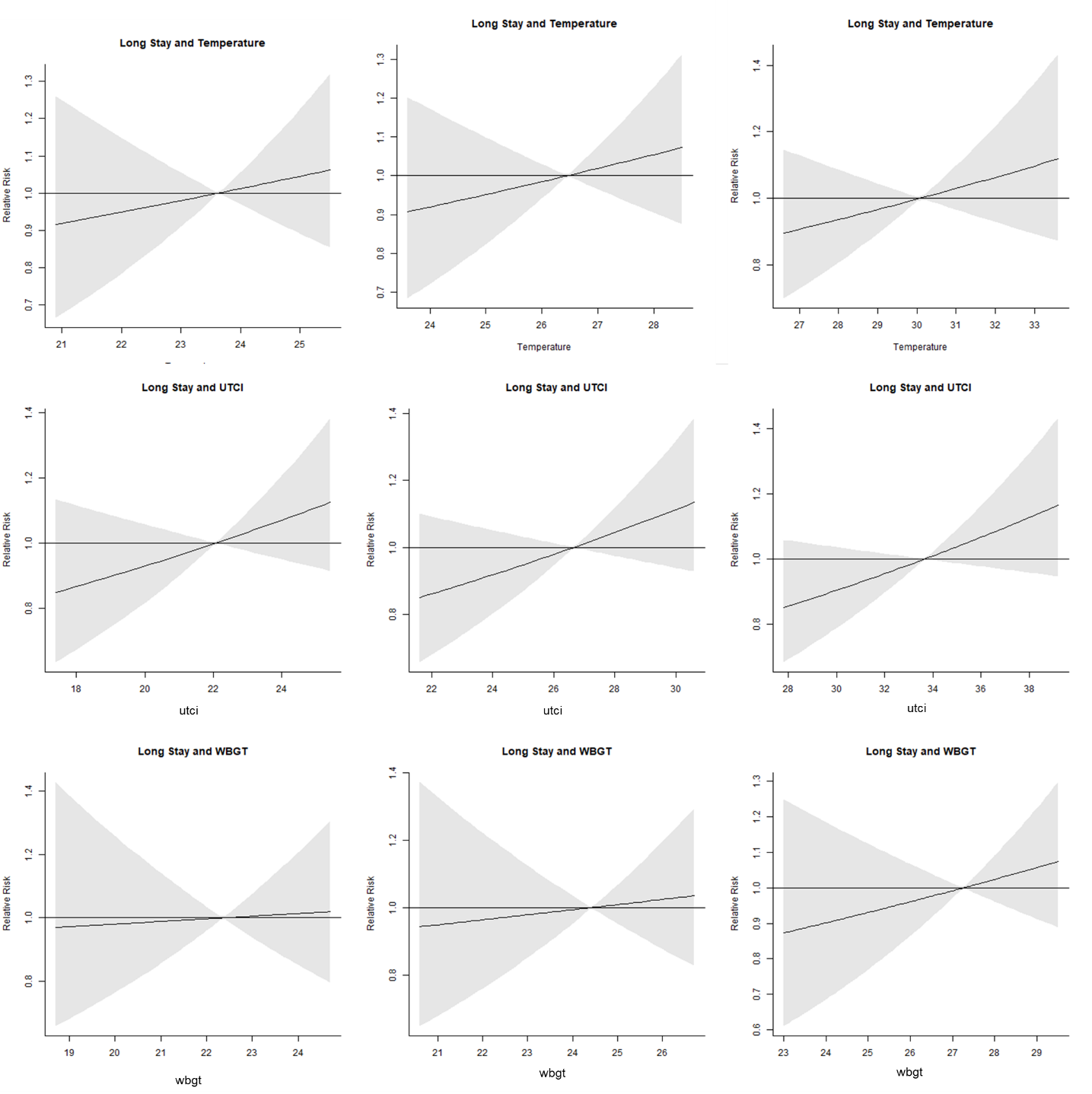


Figure S10: Relative Risk of higher percentages of Longer Stay in Hospital and exposure to heat as indicated by three heat metrics (R to L) monthly mean of daily minimum, mean and maximum temperature (top) utci (middle) and wbgt (bottom).


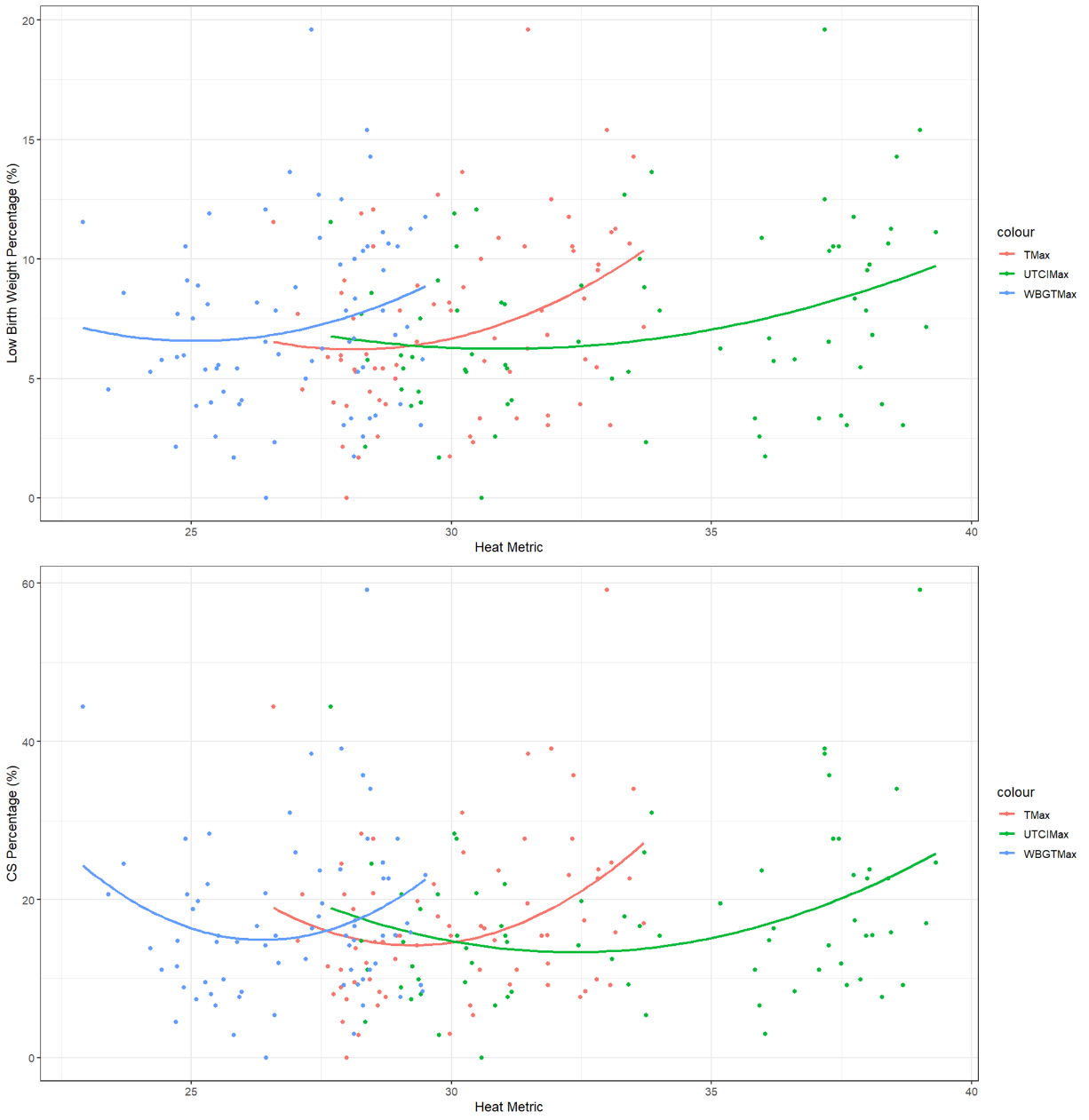


Figure S11: Distribution of the Monthly mean of the daily maximum values for the 3-heat metrics, temperature, utci and wbgt. Top plot is for percentage of low birth weight and bottom plot is for percentage of Caesarean Sections (CS).
